# Bone Morphogenetic Proteins Shape T_reg_ Cells

**DOI:** 10.3389/fimmu.2022.865546

**Published:** 2022-03-28

**Authors:** Piotr Kraj

**Affiliations:** Department of Biological Sciences, Old Dominion University, Norfolk, VA, United States

**Keywords:** Treg, Th17, BMP, BMPR1α, immunity, epigenetic, Kdm6b, Cdkn1a

## Abstract

The transforming growth factor-β (TGF-β) family includes cytokines controlling cell behavior, differentiation and homeostasis of various tissues including components of the immune system. Despite well recognized importance of TGF-β in controlling T cell functions, the immunomodulatory roles of many other members of the TGF-β cytokine family, especially bone morphogenetic proteins (BMPs), start to emerge. Bone Morphogenic Protein Receptor 1α (BMPR1α) is upregulated by activated effector and Foxp3+ regulatory CD4+ T cells (Treg cells) and modulates functions of both of these cell types. BMPR1α inhibits generation of proinflammatory Th17 cells and sustains peripheral Treg cells. This finding underscores the importance of the BMPs in controlling Treg cell plasticity and transition between Treg and Th cells. BMPR1α deficiency in *in vitro* induced and peripheral Treg cells led to upregulation of Kdm6b (Jmjd3) demethylase, an antagonist of polycomb repressive complex 2 (PRC2), and cell cycle inhibitor Cdkn1a (p21Cip1) promoting cell senescence. This indicates that BMPs and BMPR1α may represent regulatory modules shaping epigenetic landscape and controlling proinflammatory reprogramming of Th and Treg cells. Revealing functions of other BMP receptors and their crosstalk with receptors for TGF-β will contribute to our understanding of peripheral immunoregulation.

## Introduction

The major polarized Th subsets, Th1, Th2, Th9 and Th17 cells, are generated in response of CD4^+^ T cells to antigenic stimulation, co-stimulatory signals and cytokines and utilize specialized effector mechanisms to eliminate different types of pathogens (1–4). TGF-β has emerged as the cytokine controlling intrinsic activation of T cells and their antigenic responses (5, 6). In the presence of IL-4 or inflammatory cytokines, especially IL-6, TGF-β supports generation of Th9 or Th17 cells respectively (7, 8). Th cell functions are controlled by regulatory CD4^+^ T cells (T_reg_), which express the transcription factor Foxp3 (9, 10). T_reg_ cells maintain immunological self-tolerance and homeostasis but also control clinical conditions including immunometabolic and degenerative diseases, and tissue regeneration (10–13). Population of thymus derived T_reg_ cells is complemented by peripheral T_reg_ cells generated from conventional CD4^+^ Th cells which upregulate Foxp3 in response to stimulation with antigen and TGF-β (14, 15). Peripheral T_reg_ cells exhibit considerable heterogeneity and utilize specialized mechanisms to constrain inflammatory reactions in response to self and exogenous antigens (16–19). Foxp3 is essential for T_reg_ cell function, especially for its suppressive activity (9). However, T_reg_ cell lineage commitment in the thymus seems to be initiated before Foxp3 expression and Foxp3 expression does not confer all features of T_reg_ phenotype like expression of CTLA-4, lack of IL-2 expression (20–23). Recent reports indicate that T_reg_-specific epigenetic changes including DNA demethylation and histone modifications establish a pattern of T_reg_ gene expression and stability of T_reg_ cell phenotype (24–27). T_reg_-specific defects often correlate with the development of several autoimmune disorders such as type 1 diabetes, multiple sclerosis, psoriasis, rheumatoid arthritis and Crohn’s disease (10, 28–31). This includes reduced induction and homing of peripheral T_reg_ cells, alleviated or altered suppressor mechanisms and decreased stability of T_reg_ phenotype. Deficiency of T_reg_ cells caused by mutations of Foxp3 results in early onset autoimmune disease as demonstrated in Foxp3 mutant *scurfy* mice and humans with IPEX (immune dysregulation, polyendocrinopathy, enteropathy, X-linked) syndrome (32, 33). Deletion of multiple other genes affecting Foxp3 protein stability or altered epigenetic status of *Foxp3* gene locus resulted in compromised function of T_reg_ cells and were associated with autoimmune pathology (34, 35). Nevertheless, compromised function of T_reg_ cells is not always associated with their reduced frequency (36, 37). For example, signaling through the IL-27R or TGF-βR, impacted T_reg_ cell function but was not accompanied by major phenotypic or quantitative changes of T_reg_ population resulting in systemic autoimmunity (38, 39).

Uncovering what mechanisms control T_reg_ cell homeostasis become even more important when it was discovered that T_reg_ cells which lost Foxp3 expression (exT_reg_ cells) may produce inflammatory cytokines, IFN-γ and IL-17 (40, 41). While downregulation of Foxp3 may be required to alleviate suppressive effect of T_reg_ cells, allowing for effective immune responses to pathogens, in other cases T_reg_ cell instability exacerbated tissue damage and contributed to immune pathology (42, 43). T_reg_ instability also contributes to the augmentation of anti-tumor immunity (44, 45). exT_reg_ cells promoted destruction of pancreatic islets and accelerated onset of diabetes (41). In rheumatoid arthritis and EAE, pathogenic Th17 cells were shown to arise from T_reg_ cells (46, 47). In contrast, resolution of inflammation may depend on the opposite process of trans differentiation of Th17 cells into T_reg_ cells (47, 48). Thus, regulation of the Th cell lineage plasticity is critical for understanding of immune regulation and pathogenesis of autoimmune diseases (49, 50).

## Generation and Maintenance of T_reg_ Population

Multiple reports identified membrane and soluble molecules which proved essential to control abundance and fitness of T_reg_ cell population in peripheral organs and promote their suppressor function. This includes signaling through the TCR, costimulatory molecules (CD28 and CTLA-4) and cytokines receptors (18, 43, 51–56). IL-2 and TGF-β were the most studied cytokines in the context of T_reg_ cell biology. IL-2 is a key cytokine required for induction of Foxp3 in thymic T_reg_ precursors and in peripheral CD4^+^ T cells (14, 57–59). Mechanistically, Stat5 in response to IL-2 signaling binds enhancer in the *Foxp3* gene inducing its expression in the thymus (60). In peripheral T_reg_ cells IL-2 induced transcriptional program controls metabolic fitness of T_reg_ cells, sustains their survival and suppressor function and prevents autoimmunity (61, 62). Foxp3 CNS2 (conserved noncoding sequence) enhancer element acts as an IL-2 sensor by binding Stat5 and conferring stable inheritance of Foxp3 expression (63). IL-2 induced genetic program of T_reg_ cell differentiation and peripheral maintenance depend on activation of Smad3 and the presence of TGF-β (59, 64–66). While both IL-2 and TGF-β promote generation and sustain T_reg_ cells, IL-2 inhibits and TGF-β enhances generation of effector Th17 cells underscoring the importance of context dependent signaling for Th lineage ontogeny (67, 68).

Immunoregulatory role of TGF-β has been known before the discovery of T_reg_ cells (69). TGF-β provides vital signals that limit immune activation so deletion of the TGF-β1 gene in experimental mice, which abrogated TGF-β signaling in multiple T cell subsets, induced severe autoimmune inflammatory disease (5, 70). T cell specific inhibition of TGF-βRII signaling had similar outcome and precipitated systemic autoimmune disease characterized by massive activation and expansion of T cells (71, 72). Co-transfer of naive CD4^+^ T cells expressing dominant negative TGF-βRII, and T_reg_ cells, into recipient mice demonstrated that effector cells need to respond to TGF-β for the T_reg_ cells to control their activation (73). T cell specific deletion of TGF-βRII revealed that TGF-β signaling is not required for thymic development of T_reg_ cells but supports Foxp3 expression, suppressor function and sustains peripheral population of T_reg_ cells (74, 75). In summary, earlier reports supported conclusions that while T_reg_ thymic development is not affected, both T cell autonomous and T_reg_ dependent tolerance mechanisms are abrogated by elimination of TGF-β signaling in effector Th and in T_reg_ cells (75–77). The caveat of these experiments is that they relied on inhibition of TGF-β signaling in multiple T cell subsets and examined T_reg_ cells in the context of induced severe autoimmune inflammatory disease, complicating interpretation of the role of TGF-β in T_reg_ cells (6). In contrast, analysis of newborn mice with T cell specific TGF-βRI gene deletion and, inhibition of TGF-β signaling in thymic organ cultures identified TGF-β, in connection with IL-2, as cytokines essential for inducing Foxp3 expression and thymocyte commitment to T_reg_ cell differentiation in the thymus (78). However, another report defined TGF-β role in T_reg_ development to be limited to enhancing survival and protection from negative selection of thymocytes committed to become T_reg_ cells (79). This report of limited impact of TGF-β in inducing T_reg_ cell generation was questioned by demonstrating that intrathymic transfer of early thymocytes, where TGF-βRI gene deletion is induced at the double positive stage, failed to produce any T_reg_ cells, corroborating reports that TGF-β signaling is indispensable for T_reg_ lineage commitment (80). In contrast, deletion of the TGF-βRI gene in T_reg_ cells following Foxp3 expression, by Foxp3 controlled cre expression, did not decrease thymic generation of T_reg_ cells, in agreement with reports that TGF-β signaling is dispensable for T_reg_ lineage commitment (80). Moreover, abrogation of TGF-β signaling in already differentiated T_reg_ cells did not decrease proportion of peripheral T_reg_ cells, Foxp3 expression was preserved and no systemic autoimmunity was observed (39). Only aged mice suffered from local skin and gastrointestinal inflammation due to selective defect of TGF-βRI deficient T_reg_ cells to migrate, accumulate and control Th17 cell mediated responses. In contrast to Th17 cells, control of Th1 effector cells by TGF-βRI deficient T_reg_ cells was enhanced. This result suggested that TGF-β does not control overall fitness of T_reg_ cells but rather modulates their suppressor function to selectively impact different Th subsets in specific organs. Another report demonstrated that T_reg_ cell mediated production of TGF-β is necessary to prevent food allergy underscoring the importance of T_reg_ derived TGF-β in allergic responses and maintenance of immune tolerance (81).

## Bone Morphogenetic Proteins, Their Receptors and Signaling Pathways

Bone Morphogenetic Proteins (BMPs) are the largest subfamily of the TGF-β cytokine superfamily which also includes TGF-β, a founding member of the family, activins, nodal and growth and differentiation factors. BMPs were identified by their ability to induce bone differentiation (82). It is now well known that in addition of inducing differentiation of osteoblasts, bone-forming cells, BMPs control multiple cellular processes including differentiation of various cell types, adhesion, migration and proliferation and apoptosis (83, 84). They have prominent role in regulating body axes formation during embryonal development, regulate epithelial - mesenchymal transition in cancer and wound healing (83, 85–87). BMPs sustain stem cell renewal and differentiation, including tissue specific and cancer stem cells (88–90). Individual BMPs often have overlapping functions, but they can be highly specific when function as morphogens or cytokines sustaining tissue homeostasis. BMPs are highly pleiotropic cytokines which act in autocrine, paracrine and endocrine fashion determined by tissue environment and intrinsic properties of target cells (91).

Tight regulation of BMP signaling is crucial to maintain homeostasis of tissues and organs, and is achieved by controlling BMP gene expression, secretion and maturation of BMP precursors. Proteases involved in producing active, mature BMPs include furin, which is induced in activated T cells and essential for T_reg_ cell suppressor function (92). Mature BMPs are bound and sequestered by soluble (e.g. chordin, noggin, gremlin) or membrane/matrix proteins (e.g. fibrin, small leucine-rich proteins) or pseudoreceptors like BAMBI (BMP and Activin Membrane-Bound Inhibitor) (93, 94). This complex system regulates BMPs bioavailability by controlling their secretion, proteolytic maturation of BMP precursors, degradation and sequestration.

**Figure 1:**
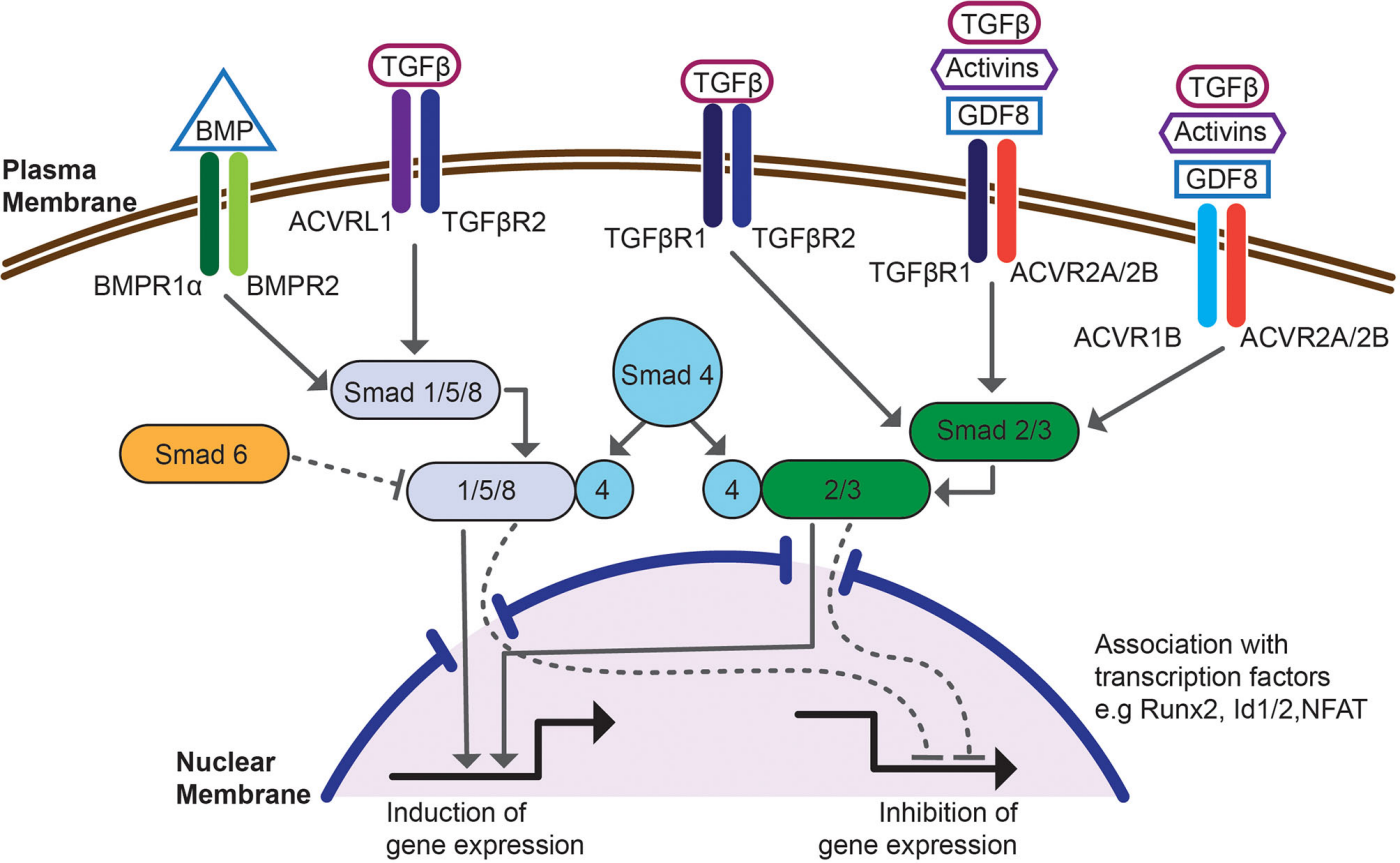
Schematic overview of the canonical, SMAD-dependent BMP and TGF-β signaling pathway. Signaling is initiated by binding to a heteromeric complex of type I receptors, e.g. BMPR1α, TGF-βR1, ACVRL1 or ACVR1, associated with type II receptors, e.g. BMPR2, TGF-βR2, ACVR2A/2B. Intracellular, BMP or TGF-β responsive transcription factors, Smads become phosphorylated and associate with co-Smad4 and translocate into nucleus. This signaling pathway is controlled by inhibitory Smad6. Once in the nucleus Smad complexes associate with transcription factors e.g. Runx2, Id1/2 or NFAT, bind regulatory regions of Smad dependent genes and regulate transcription. The figure shows interdependence of BMP and TGF-β signaling at the level of receptor binding and Smad phosphorylation.

TGF-β family cytokines, including BMPs, signal through heteromeric complexes of type I and type II receptors, which have activity of serine/threonine kinases (**Figure 1**). Cytokine ligand binds to a type II receptor and the ligand-receptor complex binds to a type I receptor. Formation of a ternary complex activates receptor kinase activity and induces phosphorylation of transcription factors, Smads, which activates canonical signal transduction pathway (84). TGF-β itself binds TGF-βRII and TGF-βRI (Alk-5) and induces phosphorylation of Smad2/3. BMPs bind one of type II receptors, BMPR2, activin receptor type 2A (ACVR2A) or activin receptor type 2B (ACVR2B). Ligand binding to type II receptor induces recruitment of one of type I receptors, activin receptor-like kinase 1 (Alk-1, ACVRL1), activin A receptor type 1 (Alk-2, ACVR1), activin receptor type 1B (Alk-4, ACVR1B), BMPR1α (Alk-3) or BMPR1β (Alk-6, not expressed by CD4 cells) and leads to conformational change of the heteromeric receptor to induce kinase activity of type I receptor and phosphorylation of Smad1/5/8. Promiscuity of ligand receptor interactions contributes to redundant functions of BMPs but also underlies signaling crosstalk between TGF-β and BMPs. TGF-β bound to TGF-βRII may recruit and transphosphorylate ACVRL1 or BMPR1α with subsequent phosphorylation and activation of Smad1/5/8 (95). Type II receptors ACVR2A or ACVR2B may also bind TGF-βRI with resulting phosphorylation of Smad2/3. Thus, combinatorial activation of both Smad pathways could be essential for signaling crosstalk of TGF-β and BMPs (96). Smad transcription factors phosphorylated by TGF-β or by BMP receptors (R-Smads) form trimeric complexes with Smad4 and translocate into nucleus. They interact with multiple co-activators, including genes controlling T_reg_ phenotype, and bind specific motifs present in regulatory regions of Smad inducible genes, including *Foxp3* (84, 97, 98). Besides activating Smads, BMPs signal through multiple Smad-independent (non-canonical) pathways (99). This involves activation of Tak-1 (TGF-β activated kinase 1) and downstream activation of NF-κB (100–104). Smad independent signaling also includes activation of PI3K-Akt pathway (105). Finally, BMPs activate p38/JNK kinases which engages TRAF4 or TRAF6 and activates Tak1 (106–108).

## Bone Morphogenetic Proteins Control of T_reg_ Lineage

While TGF-β mediated regulation of Th lineage differentiation and immune system homeostasis have been extensively studied, the role of other members of the TGF-β family, including BMPs is only starting to emerge (109). Recent reports demonstrate that BMPs, similar to TGF-β, are immunomodulatory cytokines which control differentiation and functions of immune cells impacting immune tolerance, inflammation and linage specification of effector Th cells (110). BMPs regulate thymic development of T cells, but published results remain controversial (111–115). Both thymocytes and thymic stromal cells produce BMPs and express BMP receptors. Fetal thymic cultures and signaling inhibitor studies showed that BMPs are required for early thymocyte progenitor homeostasis but block transition from double negative to double positive thymocytes (112, 116). In contrast, analysis of conditional knockout mice where BMPR1α gene was deleted in hematopoietic cells (by crossing to vav-cre mice) did not reveal changes in thymus cellularity and subset proportions (114). Analysis of mice where BMPR1α gene was deleted in double positive thymocytes showed normal development of T cells with the exception of a population of Foxp3^+^ T_reg_ cells which was severely decreased suggesting a unique role of this receptor in T_reg_ specification (117). However, thymic but not peripheral T_reg_ population was normal when BMPR1α gene was deleted at the later stage, in thymocytes expressing Foxp3 (118).

BMPR1α is expressed in mature CD4^+^ T cells in lymph nodes, spleen and peripheral organs (118). It is expressed at low level in naive CD4^+^ T cells and at higher levels in activated Th and T_reg_ cells. It is upregulated following T cell activation within hours. Since expression of BMPR2 is not affected by T cells activation, it is upregulation of BMPR1α which renders activated CD4^+^ T cell sensitive to BMPs (119). *In vitro* studies using signaling inhibitors have shown that BMPs regulate proliferation and activation of CD4^+^ T cells but the role of BMPs in controlling peripheral T_reg_ cells was not addressed (120, 121). Blockade of BMP signaling in rheumatoid arthritis patients augmented inflammation induced by IL-17 and BMPs ameliorated intestinal inflammation suggesting that cellular targets of BMP signaling may include effector Th17 and T_reg_ cells (122–125). BMP2/4 or activin A synergized with the TGF-β to generate inducible T_reg_ (iT_reg_) cells but were not able to completely replace TGF-β and induce Foxp3 expression (126, 127). Foxp3 enhancer, CNS1, contains canonical Smad1/5/8 binding site that partially overlaps Smad2/3 site. T cells activated in the presence of BMPs differentiated into Th1 or Th2 but Th17 differentiation was inhibited. BMP signaling resulted in inhibition of Rorc and IL-17 upregulation (119). These results were complemented by analysis of CD4^+^ T cells deficient in BMPR1α (117, 119). Generation of Th17 cells *in vitro*, induced by IL-6 and TGF-β, is greatly enhanced by abrogation of the BMPR1α signaling but it still requires presence of TGF-β. At the same time, *in vitro* generation of iT_reg_ cells is impaired, not improved, by BMPR1α deficiency, suggesting complex interaction between BMPR1α and TGF-β signaling pathways (117). Deletion of BMPR1α gene does not affect phosphorylation of Smad2/3 in CD4^+^ T cells activated for 1 hour in the presence of TGF-β, however genes mediating responses to TGF-β signaling, including *Smad3*, *Tsc22D1*, *Skil*, were differentially expressed when analyzed after 4 days (128, 129). Transcriptome analyses using RNA-seq revealed that of 72 transcription factors identified as differentially expressed between wild type and BMPR1α deficient iT_reg_ cells, 39 included genes identified in previous reports to support Th17 cell differentiation and 17 to support iT_reg_ cell generation (130–135). Transcription factors *Rorc*, *Rxra*, *Batf*, *Maf*, *Ikzf2* and *Ikzf4* were overexpressed in BMPR1α deficient T_reg_ cells, while *Hopx* and *Foxp3* had lower expression compared to wild type T_reg_ cells (118). BMPR1α deficient iT_reg_ cells also had lower expression of *Crem*, *Pde3b* and *Gpr83*, genes associated with T_reg_ phenotype (21, 118, 136, 137). Thus, BMPR1α signaling in naive cells affects developmental programme controlling lineage choice of iT_reg_ and Th17 cells and, likely, balance between these two cell subsets.

## Altered Ontogenesis and Phenotypic Stability of BMPR1α Deficient T_reg_ Cells

Abrogation of BMPR1α signaling in mature T_reg_ cells resulted in increased proportion of T_reg_ cells expressing low levels of Foxp3, as mice aged, and significantly altered proportions of T_reg_ cells expressing naive (CD44^low^CD62L^+^) and mature (CD44^+^CD62L^low^) phenotype. T_reg_ cells still expressing high levels of Foxp3, and naive phenotype, were replaced by cells with low expression of Foxp3, and mature phenotype, and these cells dominated peripheral T_reg_ population in aged mice. Acquisition of mature phenotype is associated with T_reg_ activation, or is evidence of cellular senescence indicating disruption of peripheral homeostasis (138, 139). Analysis of cell surface markers showed that BMPR1α-deficient T_reg_ cells expressed lower levels of CD39 and Klrg1, indicating that their suppressor function and terminal maturation are impaired (140, 141). Phenotypic changes of the T_reg_ population were accompanied by gradual upregulation of CD44, and downregulation of CD62L, on conventional CD4^+^ T cells in aging mice. Progressive loss of Foxp3 expression, associated with senescent phenotype, and increased presence of activated, conventional T cells, are consistent with compromised T_reg_ cell suppressor function and unstable T_reg_ phenotype (118).

When wild type or BMPR1α-deficient T_reg_ cells, expressing high levels of Foxp3, were co-transferred to lymphopenic mice, with naive conventional CD4^+^ T cells, only wild type T_reg_ cells retained Foxp3 expression, and were able to protect recipient mice from inflammatory bowel disease. BMPR1α-deficient T_reg_ cells had high expression of CCR6 and IL-23R, receptors regulating homing and promoting differentiation of Th17 cells or their precursors. This was associated with increased levels of Rorc, IFN-γ and IL-17 in donor BMPR1α-deficient cells (41).

Immunization of mice with BMPR1α-deficient T_reg_ cells led to robust activation of conventional CD4^+^ T cells, which expressed higher levels of activation markers, and inflammatory cytokines IFN-γ and IL-17. BMPR1α deficient T_reg_ cells in immunized mice had lower expression of Foxp3, CD39, 4-1BB, and Klrg1. CD39 is an ectonuclease directly involved in T_reg_ suppressor function, and 4-1BB binding of galectin-9 augments T_reg_ function (140, 142–144). Klrg1 is upregulated on antigen activated, highly suppressive T_reg_ cells (141). Similarly, exacerbated inflammatory response was observed in mice infected with Citrobacter rodentium, a mouse model of bacterial colitis (145). These findings indicate unstable phenotype, and decreased ability of BMPR1α-deficient T_reg_ cells, to control inflammation and point to the importance of BMPs signaling to control immune homeostasis *in situ* and in inflammation.

## Signaling Circuits Controlled by BMPR1α Signaling

**Figure 2:**
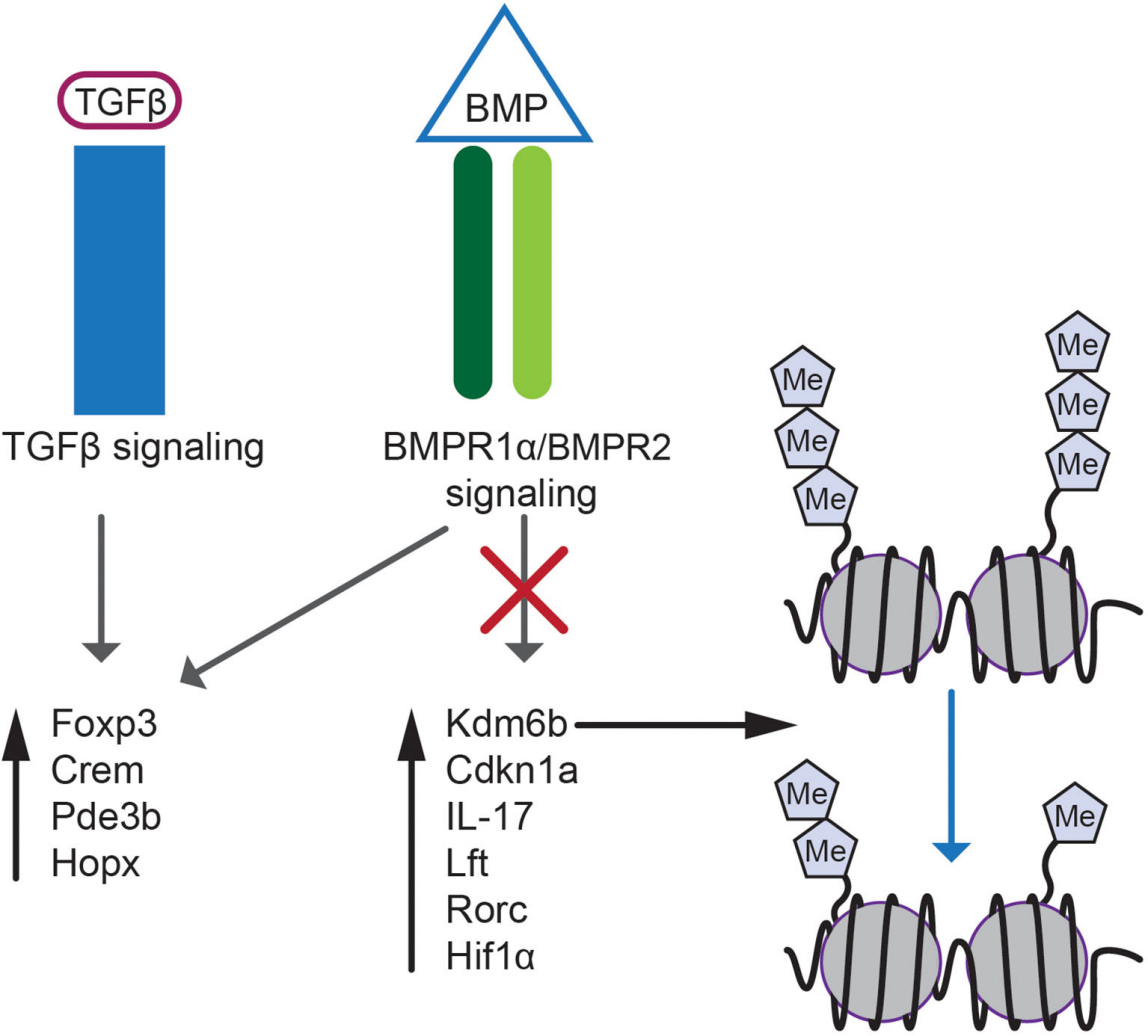
BMP and TGF-β signaling in T_reg_ cell biology. BMP and TGF-β signaling regulates expression of genes essential for T_reg_ lineage specification e.g. Foxp3, Crem, Pde3b and Hopx. Selective abrogation of BMPR1α signaling results in altered gene expression and upregulation of Kdm6b, Cdkn1a, IL-17, Lft, Rorc and Hif1α. Expression of proinflammatory genes is regulated by demethylation of inhibitory H3K27m3 epigenetic marks by Kdm6b demethylase.

Transcriptome analyses of T_reg_ and iT_reg_ cells revealed that BMPR1α gene deletion results in elevated levels of genes promoting phenotypic plasticity and functional adaptation of T_reg_ lineage cells including *Rorc*, *IRF4*, *Hif1α*, *Batf3* (**Figure 2**) (118, 146, 147). This finding is consistent with observed downregulation of *Foxp3* and enhanced production of Th1/Th17 cells in inflammatory conditions by BMPR1α-deficient T_reg_ cells (46, 148–150). In addition, a set of genes differentially expressed between BMPR1α-sufficient and deficient T_reg_ and iT_reg_ cells included *Cdkn1a* (p21^Cip1^) and *Kdm6b* (Jmjd3). Higher levels of these genes in BMPR1α-deficient cells provided cues how BMP signaling shapes T_reg_ population (**Figure 2**).

Cdkn1a is a cell cycle inhibitor associated with cell maturation and senescence (151). Higher expression of Cdkn1a in peripheral BMPR1α-deficient T_reg_ cells correlates with decreased proliferation and renewal of this subset while promoting maturation and senescence. Cdkn1a also controls CD4^+^ T cell responses to antigen and generation of memory or anergic cells (152). Kdm6b demethylase is an antagonist of polycomb repressive complex 2 (PRC2) which sustains repressive trimethylation of H3K27. Differentiation of wild type, naive CD4^+^ T cells into iT_reg_ cells is associated with downregulation of Kdm6b. In contrast, Kdm6b expression remains elevated when BMPR1α-deficient CD4^+^ T cells when they differentiate into iT_reg_ cells. Kdm6b is also elevated in T_reg_ cells directly isolated from mutant experimental mice (118). In CD4^+^ T cells Kdm6b promoted proinflammatory immune responses and enhanced cellular senescence (153). Upregulation of Cdkn1a and Cdkn2a (p16^Ink4^), controlled by Kdm6b, regulated cell cycle and inhibited reprogramming into self-renewing pluripotent stem cells supported by BMP signaling (88, 154, 155). Consistent with these reports, Cdkn1a expression in T cells was found to depend on epigenetic status of DNA and was upregulated by histone deacetylase inhibitors (156).

Mechanistic control of T_reg_ cells by Kdm6b and BMPR1α signaling is consistent with reports demonstrating that inhibition of Ezh2, a H3K27 methyltransferase of the PRC2, compromised T_reg_ cell function in tumors and autoimmune diseases (157, 158). Ezh2 is induced in T_reg_ cells upon activation, and sustains T_reg_ cell stability and function in inflammation (159–161). Deletion of Ezh2 gene in T_reg_ cells increased production of exT_reg_ cells, infiltration of CD8^+^ and effector CD4^+^/T_reg_ ratio in tumors, production of TNF-α and IFN-γ (157). Altogether, BMPR1α signaling in T_reg_ cells modulates expression of Kdm6b, an antagonist of Ezh2, and epigenetic landscape controlling T_reg_ cell plasticity.

## Discussion

Dysfunction of T_reg_ cells, resulting in altered balance between effector and T_reg_ cells, is considered a main underlying cause of most autoimmune diseases (162). Acquisition of effector Th cell functions, rather than decreased proportions of T_reg_ cells, are the main cause of autoimmune pathologies but little is understood how this process is controlled (163). Heterogeneity of the T_reg_ cell population may account for effector like properties of T_reg_ cells, while Foxp3 expression is retained (43, 164). In addition, genetic cell fate mapping, suggested that phenotypic plasticity of the T_reg_ cell lineage, especially in inflammatory environment, results in the presence of different proportions of effector CD4^+^ T cells that downregulate Foxp3 expression (41, 165). Finally, the functions of T_reg_ cells are shaped by tissue specific environmental factors, leading to the development of specialized subsets of T_reg_ cells controlling tissue homeostasis and regeneration (11–13, 166).

Foxp3 expression and development of a specific epigenetic signature are required to sustain T_reg_ functions (167, 168). Abrogation of BMPR1α signaling in T_reg_ cells led to a gradual loss of Foxp3 expression, and was associated with upregulation of transcription factors specific for effector Th lineages, Th1 and Th17 cells. Molecular changes were accompanied by decreased suppressor functions *in situ* and enhanced responses to immunization or bacterial infections. These findings are consistent with reports demonstrating that inhibition of the BMP signaling exacerbated rheumatoid arthritis, and BMPs treatment ameliorated renal inflammation (122, 125). Altered transcriptional landscape in BMPR1α-deficient T_reg_ cells was associated with epigenetic changes, mediated by overexpression of the Kdm6b demethylase (118, 153). Overexpression of Kdm6b impaired generation of iT_reg_ cells, and promoted inflammation by enhanced generation of Th17 cells (169, 170). Overexpression of Cdkn1a in BMPR1α-deficient T_reg_ cells led to acquisition of mature, senescent phenotype and decreased proliferation of T_reg_ cells. This result is consistent with ealier reports of BMPs regulating renewal and differentiation of embryonic and tissue specific stem cells including T cell progenitors (88, 115, 155). T_reg_ cell senescence may be a factor in progression of chronic autoimmune diseases (171). In summary, BMPs and BMPR1α signaling controls critical molecular circuits, impacting both Foxp3 expression and epigenetic landscape of T_reg_ cells. While little is known how BMPs may affect tissue resident T_reg_ cells, one could speculate that tight control of BMP secretion, maturation and stability predisposes them to perform immunoregulatory functions and contribute to the acquisition of organ specific features.

## Author Contributions

PK conceived the idea for the review, outlined, and wrote the manuscript.

## Funding

This work was supported in part by funding from the R03 AI159280 grant from NIAID to PK.

## Conflict of Interest

The author declares that the research was conducted in the absence of any commercial or financial relationships that could be construed as a potential conflict of interest.

## Publisher’s Note

All claims expressed in this article are solely those of the authors and do not necessarily represent those of their affiliated organizations, or those of the publisher, the editors and the reviewers. Any product that may be evaluated in this article, or claim that may be made by its manufacturer, is not guaranteed or endorsed by the publisher.
